# Achieving a High Response Rate With a Health Care Provider Survey, Washington State, 2006

**Published:** 2010-08-15

**Authors:** Nguyet Tran, Julia A. Dilley

**Affiliations:** Washington State Department of Health; Multnomah County/Oregon Department of Human Services, Portland, Orego

## Abstract

**Background:**

Growing evidence of deficiencies in patient safety, health outcomes, cost, and overall quality of care in the United States has led to proposed initiatives and conceptual frameworks for improvement. A means for feasible, valid, and ongoing measurement of health care quality is necessary for planning and evaluating such initiatives.

**Community Context:**

We sought to assess and improve health care quality for the management of chronic diseases in Washington State. We used the Chronic Care Model to develop a survey for health care providers and systems that measured quality of care and monitored improvement for multiple chronic conditions.

**Methods:**

We surveyed a random sample of primary care providers and their clinic managers. We used 2 complementary tools: a provider questionnaire (administered by mail) and a clinic manager questionnaire (administered by telephone) to measure intermediate indicators of health care quality.

**Outcome:**

We achieved high response rates (78% for physicians, 82% for physician assistants, and 71% for clinic managers).

**Interpretation:**

Our survey administration methods, or modified versions of these methods, may be effective for obtaining high response rates as part of ongoing monitoring of health care quality.

## Background

The prevalence of chronic disease in the United States is high and will continue to increase because of the aging and longevity of the population (1). Growing evidence of deficiencies and concern over gaps in health care quality (2) have led to proposed initiatives and conceptual frameworks to improve patient safety, health outcomes, cost, and quality of care in the United States. A means for feasible, valid, ongoing measurement of health care quality is necessary for planning and evaluating such initiatives.

High-quality care reflects the most current professional knowledge (3). Some models systematically measure health care quality. For example, the Healthcare Effectiveness Data and Information Set (HEDIS) is a set of standardized health care performance measures maintained by the National Committee for Quality Assurance (4). These performance measures are constructed from administrative data. However, they are not useful for describing the quality of health care received by all people at a state or regional level because HEDIS data are collected at the health plan level and only for managed care plans.

The Agency for Healthcare Research and Quality has provided an annual report describing the quality of the nation's health care since 2003, based on 45 core measures from existing data sets (3). State-level data are also available and useful, but these distal (patient-level) indicators of health care quality raise many questions about what components of health care systems are succeeding or failing.

## Community Context

In 2005, approximately 13,000 physicians and 1,500 physician assistants (PAs) served more than 6 million Washington State residents (5). The Washington State Department of Health helps to oversee health care providers and the approximately 1,500 health care facilities where they work, partly through licensing and disciplinary actions.

In Washington State, public health professionals in chronic disease programs support health care providers and practices with efforts to improve health care quality. For example, the state's comprehensive tobacco control program promotes provider training programs and supporting materials to implement clinical best practices for tobacco use screening, brief advice to quit, and referral to support resources including the state's quit line and use of pharmacotherapy. The state's asthma coalition disseminates clinical guidelines for asthma control. The state's diabetes program recruits clinics into diabetes collaboratives to improve implementation of proactive diabetes care. Some of these programs have developed close working relationships with health care systems (such as major hospital systems, health maintenance organizations, provider support networks, insurance providers, and individual providers). Other programs also have identified strategies for improving public health by supporting clinical systems change. We recognized that health care providers might become overwhelmed by uncoordinated contacts from public health programs and sought to improve the efficiency of outreach to clinical health care systems and to monitor the results of integrated initiatives.

A common framework was needed to measure health care quality by using a method that was feasible for ongoing collection to monitor improvement. The Chronic Care Model uses evidence-based interventions to transform a reactive health care delivery system into one that engages patients and those around them with the goal of maintaining wellness (6). Promising evidence shows that the Chronic Care Model and its components can be a successful framework to improve care for patients (7,8), and we used it to measure health care quality. These measures could most logically be collected by surveying providers and clinic managers, but we were concerned that a survey might not provide reliable information because of low response rates.

We measured intermediate indicators of health care quality by using a survey aligned with the Chronic Care Model. Such indicators — when examined alongside patient-level indicators of health care quality — may be useful for planning interventions and monitoring progress in health care systems change. The specific objective of this study was to test whether our survey methods could yield high response rates from health care providers.

## Methods

### Instrument development

In close consultation with public health and clinical partners, we developed questions to assess whether components of the Chronic Care Model were present in health care settings. The survey consisted of 2 complementary tools: a provider questionnaire (either physician or PA) and a clinic manager questionnaire. We pilot the final provider questionnaire with 10 providers before fully disseminating it. Overall, the survey was favorably received by pilot testers, and comments were minimal.

The provider survey could be completed on a hard copy or online. The front cover of the hard copy listed an Internet address and a personal code. Surveys asked about demographics, training, routine care for specific conditions, engaging patients to actively take a role in their own health, knowledge of resources, and use of clinical practice guidelines. Provider surveys had 135 questions either in a multiple-choice or yes/no format, except for the final question, which was open-ended to allow unstructured input.

Using a shorter, telephone-based survey, we asked clinic managers about policies and business systems that support the delivery of care to patients: medical information systems, quality improvement, and official clinical practice guidelines related to chronic disease care. The average length of a telephone interview with a clinic manager was less than 15 minutes. Clinic manager telephone surveys were administered by trained interviewers using computer-assisted software (Interviewer CATI, Voxco, Montreal, Quebec, Canada). The study was approved by the Washington State Institutional Review Board.

### Study population

We used lists from different sources to represent statewide health care systems and providers. We obtained the physician list from the Washington State Medical Association (WSMA) master list of licensed physicians in November 2005. WSMA charged a nominal administrative and per-record fee. To our knowledge, the WSMA master list contains the most complete repository of physicians' names, addresses, and specialties in the state. WSMA pulled all data for members and nonmembers representing 5 areas of medical practice: family medicine, general practice, general internal medicine, obstetrics and gynecology, and general pediatrics. These areas of practice are classified by the federal government as primary care (9).

We obtained the PA information from the Health Systems Quality Assurance Division at the Washington State Department of Health. Although the PA data did not specify the provider's area of specialty, information was sometimes available on place of employment and practice. We obtained the list after providing information about the project and assurance of its noncommercial nature.

Last, we identified clinic managers for the selected providers during the initial screening calls to providers (to verify delivery address) and from the provider survey. If a clinic did not employ a clinic manager, we asked for the name of the person who would be most familiar with the business systems of that clinic. We also contacted all clinics for which no clinic manager name was provided.

### Sampling and subject selection

We sampled physicians and PAs separately (Figure). We used a random sample to select physicians for the study, stratified by urban or rural county to ensure sufficient numbers of physicians from rural areas of the state. The WSMA file included 7,128 records indicating specialty. To obtain our goal of 500 or more completed surveys for valid statewide estimates, we assumed a 60% response rate and mailed surveys to 838 physicians.

**Figure 1.**
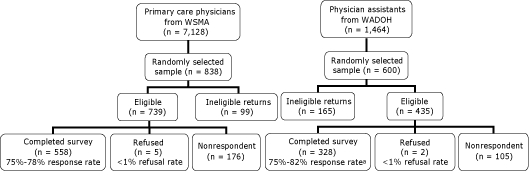
Final study disposition for physicians and physician assistants, Health Care Provider Survey, Washington State, 2006. Response rates are presented as the range of unadjusted to adjusted percentages. Unadjusted response rate is the proportion of surveys completed by total number of eligible providers; nonrespondents are included in the denominator. Adjusted response rate assumes that the proportion of nonrespondents is equivalent to the proportion of providers for whom eligibility or ineligibility could be determined. This proportion for physicians was 85% (completed plus refused divided by total sample with nonrespondents removed). We considered 85% of the 176 nonrespondents as "likely eligible" (n = 150). Therefore, the denominator for the physicians' adjusted response rate was the sum of completed surveys plus refused plus "likely eligible" (n = 713). The analogous proportion for physician assistants was 67%. Ineligible returns include those not meeting study inclusion criteria and surveys returned as undeliverable. Abbreviations: WSMA, Washington State Medical Association; WADOH, Washington State Department of Health.

We used a simple random sampling design to select PAs. Using information on place of employment (if available), we removed 65 records identifying PAs that practiced in a specialty clinic (eg, dermatology, surgery, medical imaging). A sample size of 1,464 records remained. We assumed a 50% response rate. To achieve our goal of 300 or more completed surveys for statewide estimates, we randomly selected 600 PAs.

Eligibility criteria were 1) currently practicing in Washington State and 2) seeing patients in a primary care capacity. Eligibility was reconfirmed on the first page of the questionnaire. Ineligible respondents included all providers who noted that their responsibilities were mostly administrative or research, were in training, or were retired. We designated providers who were eligible but explicitly said that they did not want to participate as refusals. Although multiple providers at the same clinic could have participated in the study, only 1 clinic manager per clinic was interviewed.

### Survey distribution

The Department of Health contracted with an independent firm to coordinate and conduct the survey administration. The initial mailings to providers occurred from January through April 2006. As described in Table 1, we sent providers up to 3 mailings over a 1-month period using a slightly modified version of the Tailored Design Method of mailed survey administration (10). The 2 modifications made to the Tailored Design Method were 1) not sending a prenotification letter to providers before mailing the survey packets and 2) using postage-paid envelopes instead of envelopes with real stamps affixed to them. As an incentive, we included a $30 check in this initial mailing of the provider survey packet. Clinic manager interviews were conducted by telephone after the survey was mailed to providers.

We contacted sampled providers by telephone before the mailing to ensure that providers were currently practicing at the listed location, to verify their delivery address, and to identify solo practices. This step was necessary to clean the sample before mailing.

## Outcome

Of the initial sample (838 physicians and 600 PAs), we obtained a total of 558 completed eligible physician surveys and 328 completed eligible PA surveys, giving us an adjusted response rate of 78% and 82%, respectively (Figure). We used the Council of American Survey Research Organizations method to calculate the adjusted response rate (11). Approximately 10% of physicians and 12% of PAs who completed the survey used the online version.

Most of the surveys were returned within the first 3 weeks of the study period (Table 1). Additional benefit was derived from a third mailing or a final contact by telephone; we received an additional 132 surveys from physicians (16%) and 175 surveys from PAs (29%) after this follow-up.

We attempted to reach 637 clinic managers (466 representing physicians and 171 representing PAs). We obtained 389 completed interviews from 589 clinic managers, giving us an adjusted response rate of approximately 71%.

Of the 838 incentive checks sent out to physicians, 660 checks were cashed. Ninety-two percent of physicians who cashed a check also cooperated in the survey (Table 2); 13% were subsequently determined to be ineligible. For PAs, 94% of those who cashed a check also cooperated in the survey; 27% were subsequently determined to be ineligible. Some physicians (n = 47) and PAs (n = 19) participated in the survey but did not cash their checks.

We did not have additional information from nonrespondents, so we were unable to directly assess whether there were important differences between providers who completed the survey and those who did not. However, to better understand the role of nonresponse bias in our study, we examined the characteristics and settings of eligible providers who participated based on the time of their responses; we considered late respondents (ie, those who did not respond to the first mailing of the survey) to be proxies for nonrespondents. This technique is standard in studies of physicians to assess survey representativeness (12). In our study, 83% (n = 463) of the 558 eligible physicians and 84% (n = 274) of the 328 eligible PAs were early respondents, who returned a completed survey within approximately the first 3 weeks. There were no significant differences in provider characteristics and practice settings between early and late survey respondents. We mailed a final report of the results to all participating providers and clinic managers who indicated interest in the results and provided a valid mailing address.

## Interpretation

Self-administered mail questionnaires can be an effective and inexpensive means of collecting epidemiologic data. However, a disadvantage that can potentially impair study validity is low response rates. Evidence suggests that response rates for mailed surveys of physicians have declined during the past decade (13). To increase the legitimacy and credibility of our study results, we used standard recommendations for enhancing cooperation: measures based on a research-driven quality framework, multimodal methods, aggressive follow-up, and a modest financial incentive. We achieved high response rates — 78% for physicians, 82% for PAs, and 71% for clinic managers — despite the somewhat complex survey (for providers, a 20-page instrument with 135 questions).

Monetary incentives significantly increase response rates both in provider populations (14,15) and in the general public (16), and in randomized trials prepayment is superior to promised payment or no incentive (17,18). Given the evidence on the effects of token incentives on responses to surveys, we included a $30 check in the initial mailing of the provider survey packet. The amount was chosen based on a previous survey conducted in the state with physicians. However, evidence suggests that even a small token financial incentive (as little as $1) can significantly improve response rates among physicians (19). We did not use a financial incentive for the clinic managers and still achieved a high response rate; however, their survey was shorter, telephone-based, and less complex than the provider survey.

Our findings are consistent with previous research and reviews that had identified modest to no significant differences between early and late respondents (12,20). Taken together, our study and these previous studies suggest that response bias may not seriously affect findings when a threshold (perhaps more than 50%) response rate is achieved. If resources are limited and prevent aggressive follow-up, a provider survey with a lower response rate may still yield representative results.

Because this was not an experimental study designed to evaluate individual strategies for improving response rates in mailed surveys of health care professionals, we were unable to evaluate how each of the steps in our approach influenced the overall response rate. Nonetheless, we were able to show that it is possible to obtain robust responses from health care providers and their clinic managers in Washington State. For this project, motivation for providers to respond may have been influenced by the incentives, design, or both (eg, salience of the topic, questionnaire design, attractive packaging of the survey form, research sponsorship).

Providers who cashed their incentive checks but did not respond were more than balanced by those who responded without cashing their checks. Physicians who refused or did not respond were paid $1,860, compared with a value of $1,770 for uncashed checks to respondents. The value of uncashed checks from PA respondents was $1,890, more than double the $780 paid to PAs who refused or did not respond.

Provider survey costs (inclusive of labor, supplies, and $30 check incentive) totaled approximately $100 per completed provider survey. Clinic manager interviews cost $30 per completed survey and no incentive was provided. We were able to obtain data for many aspects of the Chronic Care Model by asking the clinic managers alone (eg, use of electronic medical records, clinic-level use of clinical practice guidelines, activities that monitor population-based quality improvement). To minimize cost, surveillance of health care quality could rely mainly on information from clinic managers, if the surveys are short and not a burden to them and if clinic managers are aware of initiatives to improve health care quality.

A high response rate does not ensure the validity of the questionnaire. However, if the measures developed for the survey did not make sense to providers, they would not have been as motivated to participate and we would have received negative feedback in the open-ended final question on the surveys. In fact, neither was the case. A debriefing with a provider advisory group to discuss the findings of the survey and whether the results captured the intent of the survey would be useful.

Our methods allowed us to achieve a high response rate from providers selected for a health care quality survey. Our approach, or modifications of our approach, may be effective for ongoing monitoring of health care quality.

## Figures and Tables

**Table 1:** Achieving a High Response Rate With a Health Care Provider Survey, Washington State, 2006

**Survey Step**	Timing and Description	No. of Completed Surveys From Physicians (%)^a^	No. of Completed Surveys From Physician Assistants (%)^b^
**First mailing**	Day 1	247 (30)	131 (22)
Contents	Copy of questionnaire booklet with original subject ID number, cover letter, $30 check incentive, postage-paid business return envelope.		
Features/personalization	Survey packet delivered using express mail with the Washington State Department of Health as sender; visually appealing and easy-to-comprehend questionnaire booklet; cover letter addressing provider by name, on official organization letterhead, and signed by the Washington State health officer using a digital (preprinted) signature; inclusion of a token financial incentive; postage-paid return envelope.		
**Second mailing**	Day 8	283 (34)	189 (32)
Contents	Postcard		
Features/personalization	Reminded providers to complete and return the survey. The postcard had the official state logo on it and a telephone number to call with questions or for a new questionnaire booklet.		
**Third mailing**	Day 18-22	46 (5)	119 (20)
Contents	Copy of questionnaire booklet with original subject ID number, reworded cover letter, postage-paid business return envelope.		
Features/personalization	Survey packet delivered using express mail with the Washington State Department of Health as sender; visually appealing and easy-to-comprehend questionnaire booklet; cover letter on official organization letterhead addressing provider by name, mentioning the check incentive in the initial mailing, and signed by the Washington State health officer using a digital (preprinted) signature; postage-paid return envelope. Sent only to providers who had not yet responded.		
**Final contact by telephone**	Day 30	86 (10)	56 (9)
Contents	Minimum of 2 attempts to speak directly with the provider or leave a message on his or her voicemail.		
Features/personalization	General reminder to complete the survey, along with a telephone number to call with questions, to request a fax, or to obtain an extra copy of the survey. Telephone calls made to only those who had not yet responded.		
**Total**		662 (79)	495 (83)

a Unadjusted sample returned was calculated as the proportion of returned surveys (regardless of eligibility) by total number of surveys sent out to physicians; nonrespondents were included in the denominator (n = 838).

b Unadjusted sample returned was calculated as the proportion of returned surveys (regardless of eligibility) by total number of surveys sent out to physician assistants; nonrespondents were included in the denominator (n = 600).

**Table 2:** Use of Incentives by Physicians (n = 838) and Physician Assistants (n = 600), Health Care Quality Survey, Washington State, 2006

**Provider **	Eligible and Returned Survey, n (%)	Ineligible and Returned Survey, n (%)	Did Not Respond, n (%)	Returned Incomplete Survey, n (%)
**Physicians**				
Cashed incentive check	511 (92)	87 (88)	58 (33)	4 (80)
Did not cash incentive check	47 (8)	12 (12)	118 (67)	1 (20)
**Physician assistants**				
Cashed incentive check	309 (94)	121 (73)	24 (23)	2 (100)
Did not cash incentive check	19 (6)	44 (27)	81 (77)	0
